# Special educators’ intentions towards supporting practice of inclusive education for students with disabilities in secondary schools in Ghana

**DOI:** 10.4102/ajod.v11i0.875

**Published:** 2022-03-31

**Authors:** Maxwell P. Opoku

**Affiliations:** 1 Special Education Department, College of Education, United Arab Emirates University, Al-Ain, United Arab Emirates; 2 Faculty of Education, University of Tasmania, Launceston, Australia

**Keywords:** inclusion, teachers, special educators, Ghana, secondary schools

## Abstract

**Background:**

Although teacher training institutions have introduced courses in inclusive education to equip teachers with the necessary pedagogical skills to teach in diverse classrooms, it has been argued that the services of special educators are essential when it comes to teaching students with disabilities in regular classrooms. Unfortunately, there is scant literature on the views of special educators regarding the enactment of inclusive education in sub-Saharan African countries, such as Ghana.

**Objective:**

In an effort towards promoting inclusive education in Ghana, there has been deployment of special educators across Ghana to supervise the implementation of inclusive education in schools. The purpose of this study was to explore the intentions of special educators towards supporting teachers to teach students with disabilities in secondary schools.

**Method:**

Ajzen’s theory of planned behaviour guided the development of interview guide for data collection for this qualitative study. Twelve special educators were purposively selected and interviewed from five districts in Ghana.

**Results:**

The participants expressed their unpreparedness to work in secondary schools because of multiple factors, such as their job description, resistance from teachers, and inadequate teaching and learning materials.

**Conclusion:**

This study concludes on the need for policymakers to reconsider the deployment of special educators to cluster of schools or geographical areas in order to supervise the education of children with disabilities.

## Introduction

The contribution of special educators towards implementation of inclusive education cannot be overemphasised. Special educators are functional leaders in schools who supervise the practice of inclusive education and promote the well-being of all students, including students with disabilities (Angelides, Antoniou & Charalambous 2010; Liasidou & Svensson 2014; Maher 2018; Poon-McBrayer & Wong 2013; Struyve et al. 2018). For instance, special educators collaborate with classroom teachers to identify the needs, strengths and weaknesses of students with disabilities, and the best practices that promote the teaching of these students (Devecchi et al. 2012; Maher 2018; Whalley 2018). All these processes are documented in the individualised education plan of students, prepared by special educators, which is a reference source for teachers and teaching aides or assistants (TAs) who are recruited to assist students with disabilities in the classroom. While there are much literature on the contribution of special educators to the successful implementation of inclusive education in advanced countries, such as Australia, Canada and the USA (Devecchi et al. 2012; Hedegaard-Soerensen, Jensen & Tofteng 2018; Lyons, Thompson & Timmons 2016; Sharma & Salend 2016; Whalley 2018), there are scant literature on their contribution in developing countries, such as Ghana.

Inclusive education is defined narrowly as creating opportunities for students with disabilities to participate in regular schools that are in their local community (Sharma et al. 2017). Globally, there are still barriers to the successful participation of students with disabilities in regular schools (see, for example, Ainscow & Sandill 2010; De Boer et al. 2011; Sharma et al. 2013, 2017). In particular, the inability of students with disabilities to participate in regular classroom activities has been attributed to a lack of skilled teachers (Conrad & Brown 2011; McKay 2016; Pearce, Gray & Campbell-Evans 2010; Sharma et al. 2013). This has prompted discussion on the need for schools to have special educators in an effort to promote the participation of students with disabilities in regular classroom activities (Liasidou & Svensson 2014; Whalley 2018). While special educators develop policies and learning plans for students with disabilities (Angelides et al. 2010; Liasidou & Svensson 2014; Poon-McBrayer & Wong 2013), teachers may provide more attention to developing lessons that take the needs of all students into consideration. Once these arrangements are in place, it is likely that all students will participate effectively in classroom activities.

Although the presence of special educators in schools helps to promote an inclusive culture, a few studies conducted in secondary schools have reported a poor working relationship between teachers and special educators (Al-Natour et al. 2015). One of the core duties of special educators is to engage teachers regarding the learning needs of students with disabilities (Devecchi et al. 2012; Hedegaard-Soerensen et al. 2018; Lyons et al. 2016). However, studies have reported the inability of special educators to visit classrooms in order to ensure that students with disabilities are being provided with adequate learning services (Al-Natour et al. 2015; Sharma & Salend 2016). This is attributed to the enormous administrative duties that special educators perform, which confine them to the office and leave them unaware of developments in the classroom. In some instances, it has been reported that principals interfere with special educators in the performance of their duties. For example, special educators are not provided the necessary financial resources or authority to execute their functions in the classroom (Struyve et al. 2018; Timberlake 2018).

With the emphasis being made on academic excellence in especially secondary schools, very little funds are allocated to special educators to purchase learning materials required to support students with disabilities (Maher & Macbeth 2014). Other previous studies have found that unavailability of funding for inclusive education explains the absence of teaching materials and recruitment of teacher aides who will support students with disabilities in the classroom (Maher 2018; Struyve et al. 2018; Timberlake 2018; Whalley 2018). It is evident that most of the above studies have relied on the accounts of special educators in terms of practices in primary schools. Thus, the body of literature on the views of special educators regarding the implementation of inclusive education in secondary schools is very small.

In the Ghanaian context, there is scanty information on the contribution of special educators towards the implementation of inclusive. As part of larger projects, it has emerged that special educators are rarely available to support the teaching of students with disabilities (Mantey 2017; Pearce et al. 2010; Poon-McBrayer & Wong 2013; Sharma et al. 2013; Singal et al. 2015). The study reported here forms part of a larger mixed project, which attempted to understand the intentions of stakeholders (teachers, school leaders and special educators) towards implementation of inclusive education for students with disabilities in secondary schools in Ghana (Opoku et al. 2020, 2021a, 2021b, 2021c). In the first phase of the study, teachers received less support from subjective norms to enable them to perform their teaching duties in the classroom (Opoku et al. 2021b, 2021c). Indeed, school leaders confirmed the inability of teachers to teach students with disabilities in secondary schools (Opoku 2021). However, one thing that came out strongly was the absence of special educators in secondary schools as an impediment to implementation of inclusive education (Opoku 2021). In an effort to practise inclusive education at all levels of education, there is a need to extend the literature and develop a comprehensive understanding of special educators’ perspectives on practices in secondary schools in Ghana. The purpose of this study was to explore the practice and preparedness of special educators to support the implementation of inclusive education in secondary schools in Ghana. This study may provide useful information on the current state of implementation of inclusive education in secondary schools, which may inform education reform and school practices.

## Study context

Ghana is located in West Africa with a population of about 30 million (Ghana Statistical Service 2021). Education is at the forefront of national development, as there are policies in place to ensure the participation of all persons in education (Ministry of Education 2016). As a result of systemic barriers present against individuals with disabilities, such as negative attitudes towards them and outright rejection of them in a society (Anthony 2011; Dogbe et al. 2019; Opoku et al. 2017, 2019), policies have deliberately been put in place, such as implementation of inclusive education, to bridge the gap between individuals with disabilities and greater society (Ministry of Education 2016). Inclusive education was formally introduced by the Government of Ghana at primary schools in selected districts during the 2003–2004 academic year (Opoku et al. 2015, 2017). Ghana’s endorsement of Salamanca Conference on Special Education (UNESCO 1994) and ratification of the UN Convention on the Rights of Persons with Disabilities (UNCRPD 2006) in 2012 are key milestones towards practising inclusive education. In 2015, an Inclusive Education Policy document was formally promulgated to guide inclusive practices (Republic of Ghana 2015), which safeguards the right of students with disabilities to access all levels of education. Accordingly, secondary schools are expected to have resources and personnel to support students with disabilities.

The need to extent inclusive education to secondary schools has received local and international support. At the international level, the United Nations (2015) has spearheaded campaigns for the alleviation of global poverty by 2030. In education, one of the cardinal pillars was the expansion of universal access to education from primary school to secondary school (United Nations 2015). In response, the Government of Ghana has made secondary school education free in order to enable all students to have access. This move of the government has coincided with global and national attempts to practise inclusive education. This probably suggests that all students including students with disabilities will be able to have access to secondary school education.

With the commitment of the Government of Ghana to instill inclusive education in the education system, the universities and colleges of education in Ghana decided to introduce courses in special education in order to prepare teachers for inclusive practices (Nketsia & Saloviita 2013; Nketsia, Saloviita & Gyimah 2016). Also, a 4-year bachelor’s degree programme was introduced in special education at two public universities, and graduates from this programme are employed by the Special Education Division (SPED) as special educators to promote inclusive education in communities and schools. In every region and district, there is a special education coordinator who oversees the implementation of inclusive education. The special education coordinators also supervise the work of the special educators in the schools. The special educators (who perform similar roles to teacher aides) are expected to work alongside teachers, supporting students with disabilities in the classroom, in a number of schools.

The practice of inclusive education appears to have stalled in Ghana. This has been attributed to factors such as limited infrastructure (Mantey 2017), inadequate teacher skills (Anthony 2011; Mprah et al. 2016; Okyere, Aldersey & Lysaght 2019), and lack of teaching and learning materials in schools (Opoku et al. 2015; Singal et al. 2015) to enhance inclusive practices. Although many studies have reported the barriers faced by schools in efforts to implement inclusive education in Ghana (Mprah et al. 2016; Okyere et al. 2019; Opoku et al. 2015), a few studies, as part of larger studies, have explored the perceptions of special educators regarding practising inclusive education. For example, it has been reported that teachers do not receive support from special educators deployed to assist them in the classroom (Opoku et al. 2015). This was because of the limited number of special educators and the lack of funds available to them for transportation to various schools to assist teachers and students with disabilities (Mprah et al. 2016). Additionally, the author and colleagues found that special educators are not provided the required teaching and learning materials to enable them to perform their duties (Opoku et al. 2015). Consequently, they seem not to make any useful contribution to the practising of inclusive education. However, these studies were limited to the experiences of special educators in relation to supporting inclusive practices in primary schools.

## Theoretical framework

As a result of complexities surrounding the implementation of inclusive education (Opoku et al. 2021c), Ajzen’s (1991) theory of planned behaviour (TPB) was adopted as a useful framework to situate this study. The TPB is an extension of the theory of reasoned action, which explains intention to perform a behaviour as an outcome of two beliefs, namely behavioural and normative beliefs (Ajzen & Fishbein 1977). While behavioural beliefs refer to an individual’s assessment of the outcome of a given behaviour, normative beliefs refer to the support or approval an individual receives from social pressure in the execution of a function. However, Ajzen challenged this two-belief proposition and suggested that there could be a third belief, known as control beliefs, which may have a direct or an indirect effect on behaviour (Ajzen 1991, 2011). Here, control beliefs refer to an individual’s confidence in his or her capacity to perform a behaviour. According to Ajzen’s view, individuals’ capacity, as well as information accessible to them, could have an impact on them. Consequently, Ajzen (2011) argued that individual’s intention to perform a given behaviour is as a result of three interconnected beliefs, namely behavioural, normative and control beliefs. These related beliefs accumulate into determinants having an impact on intentions, which are the antecedent of behaviour.

The related beliefs accumulate into determinants of intentions (Cooke et al. 2016). For instance, the beliefs develop as follows: behavioural beliefs develop – attitude towards a behaviour; normative beliefs – subjective norms, and control beliefs – perceived behavioural control, referred to as self-efficacy in the previous inclusive education research (see Ahmmed et al. 2014). In this study, the determinants of intentions are operationally defined. Firstly, attitudes towards inclusive education are referred to as perceptions of other stakeholders, such as teachers and school leaders, towards practising inclusive education and the role of special educators. Secondly, subjective norms are referred to as pressure or support from significant others to special educators towards the practice of inclusive education. Here, consideration was given to assistance from school leaders, the SPED and the government, and parents towards practising inclusive education. Thirdly, self-efficacy refers to confidence and availability of resources to assist special educators and teachers.

Recently, studies on inclusive education have been adopting the TPB to assess intentions towards implementation of inclusive education (e.g. Ahmmed, Sharma & Deppeler 2014; Opoku et al. 2021c; Yan & Sin 2014). However, many of these studies were limited to assessment of teachers’ intentions only. As inclusive education requires a substantial contribution from diverse stakeholders, such as special educators, it is critical to develop a holistic understanding of the views of special educators, whose services have been argued as being pivotal in efforts to practise inclusive education (Liasidou & Svensson 2014; Lyons et al. 2016; Poon-McBrayer & Wong 2013). This study was guided by the following research question: ‘How prepared are special educators to support the implementation of inclusive education in secondary schools in Ghana?’.

## Method

### Participants

Participants (*N* = 12) for this study included special educators recruited from five districts (Ejisu-Juaben municipal district, Kumasi Metropolis, Mampong municipal district, Obuasi municipal district and Sekyere south district) in the most populous region of Ghana, namely the Ashanti region (Ghana Statistical Service 2012). The study area was selected because it is one of the regions selected to pilot inclusive education in Ghana. The inclusion criteria were as follows: (1) qualified special educator, (2) working in the study area and supervising implementation of inclusive education, and (3) consented to take part in this study. All the special educators (*N* = 15) deployed to support the implementation of inclusive education in the study areas were invited and those who agreed to take part in this study were recruited.

All participants (*N* = 12) had at least a bachelor’s degree in special education (see Table 1 for demographic details). While three participants were special educators playing supervisory and sensitisation roles, such as advocating for the inclusion of children with disabilities in regular classrooms (coordinators), of the special educators nine worked as teacher assistants (TAs) who are also called resource teachers in Ghana.

**TABLE 1:** Demographic characteristics of participants.

Categories (*N* = 12)	Frequency
**Groups**	
Special educators as teacher aides (TAs)	9
Special educators as coordinators	3
**Age (years)**	
26–35	2
36–45	8
≥ 46	2
**Gender**	
Male	7
Female	5
**Education status**	
Bachelor’s degree	10
Master’s degree	2
**Work experience (years)**	
≤ 5	1
6–10	1
11–15	3
16–20	4
21	3

### Instrument

An interview guide was developed based on components of the TPB. The interview guide covered the following areas: special educators’ intentions, attitudes towards inclusive education, support from significant others and self-efficacy of special educators towards practising inclusive education (see Appendix 1). The interview guide was piloted on three graduate students with many years of experience teaching in inclusive schools. They provided feedback on the tool, which was discussed with other experts, whose views were incorporated into the final draft used for data collection.

### Procedures

Of the 15 special educators in the region that were invited to take part in the study, three declined, because they were involved in other assignments outside the region at the time of data collection. Arrangements were made for face-to-face interviews to be conducted with those who agreed to take part in the study. The interviews were conducted in offices or schools at a time convenient for the participants.

Data were collected over a 6-week period between January 2018 and February 2018. The duration of the interviews ranged from 30 min to 3 h. The objective of this study was explained to all participants, who signed written informed consent forms before the interviews. Participants were informed of their right to withdraw from the study at any time without consequences. They were informed that their decision not to take part in the study would not affect their relationship with the author, the Ghana Education Service (GES), or the SPED. They were told that neither their identity nor the area of work would be disclosed to anyone outside the research team. Descriptors and sequence of interviews were used to describe the study participants. While participants working in classrooms were referred to as TAs, those working outside the classroom and supervising the implementation of inclusive education were called coordinators. All interviews were conducted in English by the author and were recorded using an audiotape, with permission from participants.

### Data analysis

The author transcribed the recorded interviews verbatim. After the transcription, the data were sent to the participants for review, so that they could advise if their responses had been captured correctly. Of the 12 participants who were contacted by email, only five responded and made suggestions, which were incorporated in the final draft. Phone calls were placed to the other participants to discuss key themes that emerged in the interviews, and they consented to their use in the study.

**FIGURE 1:**
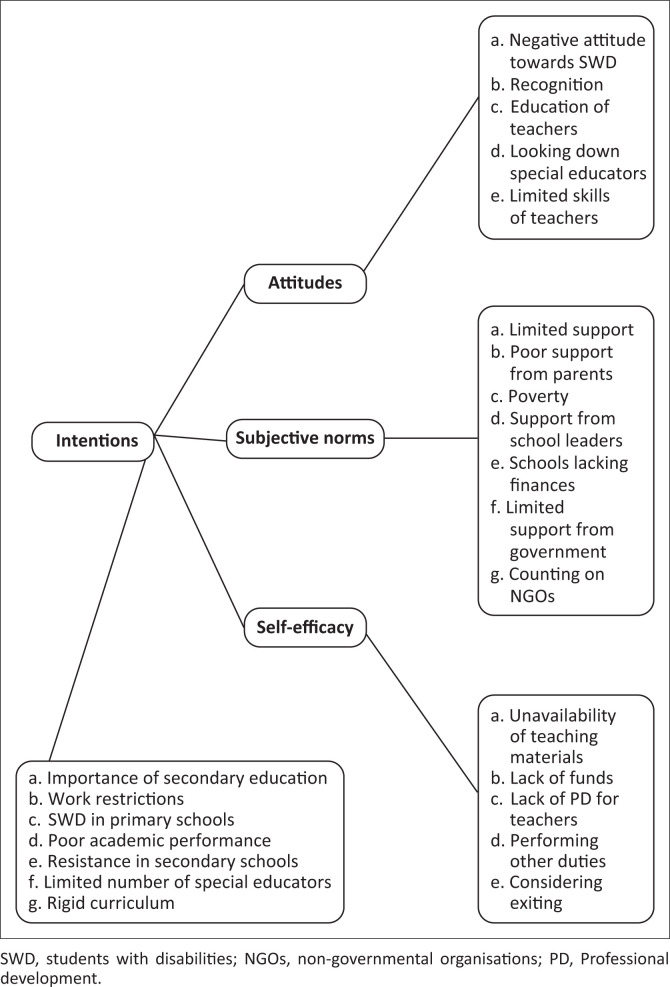
Summary of themes and categories.

As the study was guided by a theoretical framework, thematic analysis, following the guidelines proposed by Braun and Clark (2006), was performed. The steps followed were as follows: reading the transcripts to familiarise oneself with the data, coding, developing categories, theme mapping and development, and drafting the analysis. It is important to state here that the TPB variables were used as *a priori* themes (intentions, attitudes, subjective norms and self-efficacy). To expand, the author read the transcribed data several times and wrote down phrases to be used as codes. At this stage, a meeting was organised between the author and an expert in qualitative research to discuss the framework and categorisations of the data under the *a priori* themes. Consensus was reached on the ideas brainstormed during the meeting. The author continued to code all the interviews and developed a coding framework, which was shared with the expert. They discussed the content and reached consensus on the framework. At this stage, the author categorised the codes with common descriptors and noted the similarities and differences between the participants. The descriptors were tabulated under the *a priori* themes (see Figure 1). The themes and associated descriptors were transferred into a Word document, and associated texts were extracted from the data. Another meeting was held between the author and the expert to discuss the content. The author developed the story line and ran commentaries on the data. The first draft of analysis was shared with the expert for feedback, which was incorporated in the write-up.

### Ethical considerations

The study and its protocols were approved by the Human Research and Ethics Committee of the University of Tasmania (reference number: H0016994). Subsequent approvals were sought from the SPED (a body supervising the implementation of inclusive education), directors of education and school principals. To elaborate, the SPED provided the author a formal letter that was addressed to the regional director of education. The letter indicated the importance of the study and urged the director to support the author with data collection. The regional director provided the author another letter that was addressed to all district directors and principals, informing them about the study and encouraging them to participate in the study. After the necessary permissions had been granted, all special educators working in the region were invited to take part in the study.

## Results

Participants revealed that limited provision has been made for them to work in secondary schools. Participants related that they are unprepared to extend their services to secondary schools. The findings are organised under the following themes: attitudes, subjective norms, self-efficacy and intentions.

### Attitudes

Participants related the attitudes of teachers towards inclusive education and the work of special educators. Many participants (*n* = 8) said that teachers and principals hold positive attitude towards teaching students with disabilities in regular classrooms. Although there are negative attitudes towards persons with disabilities in a society, participants who work in junior secondary schools revealed that principals and teachers include students with disabilities in their lessons. Participants stated that many teachers have taken courses in special education during their pre-service training. These courses have exposed teachers to implementation of inclusive education and how to support students with disabilities to perform at their best:

‘Oh, now the teachers understand that the students with learning disabilities are supposed to be in regular classrooms, and they support them even if I’m not around. I can say that we have made progress in this regard. When I came, I invested time and energy to educate the teachers so they accept every student.’ (Female, Teacher aide [TA]1, Abena)

‘They are being accepted in schools. Principals and class teachers now accept that they have to treat them as any other member of their class. What is left is the resources that is needed for smooth implementation of the program.’ (Male, TA4, James)

Conversely, three participants who worked in an integrated unit (a special classroom in a regular school) related that some teachers still hold negative attitudes towards students with disabilities. They mentioned that those teachers do not want to accommodate students with disabilities in their lessons:

‘Within our own compound [*school*], we can’t educate the teachers who are here. Some of the teachers are not interested in education. They would tell you it’s not their duty to teach students with disabilities. They don’t listen to our advice.’ (Female, TA10, Gifty)

‘Some have negative attitudes, because they have misconceptions about these children. I see how they talk to students with disabilities when they go close to them. When some of the children go to their classroom, they smack them with canes. This is a school community, and we are one, and they [*students*] are supposed to learn together.’ (Male, TA12, Nana)

Although half of the participants claimed that they have a cordial relationship with other staff members, three participants said that the regular classroom teachers have negative attitudes towards them. Some participants revealed that their colleagues understand their work, while others said that their colleagues look down on their work:

‘They are happy with the work we are doing. Some call me to ask why I’ve not been coming to their school if they have not been there for long. I remember not going to a school for some time, and the headmaster called and asked when I’m coming to his school to support the staff.’ (Female, TA1, Abena)

‘The teachers tell me my work is unnecessary, because with the implementation of inclusive education each school should get a resource teacher. As a person moving from school to school, they think what I’m doing is not all that necessary. Unless we have resource teachers in all schools, they [*teachers*] won’t respect us. I will recommend at least two or three resource teachers for every school.’ (Male, TA3, Emma)

Almost all participants (*n* = 11) admitted that regular classroom teachers do not have the requisite skills to teach students with disabilities. Although they have taken courses in special education, the courses are not sufficient for them to teach all students, as they are unable to include students with disabilities in the absence of TAs:

‘But I think the semester course is not enough. Our education system is all about examination. So the teachers passed examination and forget everything they have been taught. This is not enough if we really want to practice inclusive education.’ (Female, coordinator 2, Felicia)

‘When you go, they will tell you to come and see some of your children. They see me as if I have the solution. But I always tell them that they can do something to help. I’m the only person catering for all the 17 schools, so I can’t move from school to school.’ (Male, TA3, Emma)

### Subjective norms

Participants mentioned the support various stakeholders provided to them when it comes to implementation of inclusive education. Generally, participants (*n* = 10) said that they do not receive much support to perform their duties as expected. Participants related that the ‘weakest link’ in the implementation of inclusion education is the parents of children with disabilities. They revealed that many parents are unconcerned about the education of their children with disabilities. Parents make limited attempts to provide their children with the necessary learning materials, food and clothing. While some parents may be willing to provide, many participants (*n* = 6) said that poverty makes it unlikely that parents will support the education of their children:

‘There was one boy that we needed to take him to rehabilitation centre for him to get vocational training. I never heard from the parents again, and I can’t be doing that work with my own money. I gave up on him, but he is still in the school. He will just pass through the system without writing any exams that will take him to the next level.’ (Female, TA1, Abena)

‘When I visit a particular school, I have to give the children money for food. Some of the parents think children with disabilities can’t grow up and be useful in societies, and because of this they don’t want spend money on them.’ (Male, TA4, James)

Many TAs (*n* = 7) related that they receive limited support from school leaders to facilitate their activities. Participants acknowledged that primary and some junior secondary school leaders allow them to work in their schools, but that they are not given any assistance for their work. Although money is released by the government to school leaders to purchase teaching and learning materials, three participants said that the government does not make any provision for purchasing of materials for students with disabilities. However, some participants (*n* = 4) commented that they understand the inability of principals to support them, as their schools do not charge fees:

‘At the beginning of every term, we submit our budget and things needed to support the teaching of students with disabilities to the school authorities. They tell us this is a regular school, and [*that*] provisions are not made for students with disabilities. I have never understood that logic. We are supposed to be considered, but they think we should also write to the education office for funds.’ (Female, TA10, Gifty)

‘Oh, they are in support of it. Where I am now the headmaster is very passionate about the policy, but there is nothing he can do to support us. It’s a public school, which is free, so they don’t charge any fees, and the government has not been giving them money to run the schools. The headmaster here at times use his own money to buy books for the children.’ (Female, TA8, Rose)

Regarding the support from government, participants felt that they do not receive any assistance for their work. They (*n* = 12) said that the government has sent them into the field to assist with implementation of inclusive education without the necessary logistics to perform their duties. Despite submitting several reports to the government through the SPED, they are yet to receive feedback on their requests. Consequently, they have resorted to non-governmental organisations (charity organisations) for finances to procure teaching and learning materials:

‘They have developed a beautiful inclusive policy document. Ask them ‘[w]here are the logistics to implement the policy?’ It is not about paper; it is about action. There was a time I was on radio and someone called in and said we come on air and make noise, but when they go to the schools, they don’t want to admit their children. The person is right.’ (Female, coordinator 5, Dora)

‘They assured us that the government is making plans to reform all schools to implement this policy. But nothing has happened, and we the resource teachers are losing interest in this work. They only pay lip service to this program and its implementation.’ (Male, TA7, Ray)

### Self-efficacy

All the participants mentioned that they have low confidence to practise inclusive education. Specifically, they have been unable to perform their duties as expected because of the unavailability of teaching and learning materials. Although participants said they have the skills and the desire to support implementation of the programme, they claimed that policymakers are yet to prioritise inclusive education, as they make little budgetary allocation for it. Participants also said that they are not provided funds to move from school to school to perform official duties. For instance, TAs are supposed to work in many schools; however, they have limited themselves to only a few schools, because they cannot afford transportation costs. Some admitted working in one school only, as they do not have funds to move around to work.

All the participants mentioned that teachers lack the confidence to support students with disabilities in their classrooms, which they attributed to a lack of pedagogical skills to teach students with disabilities. Lack of funds was cited as a barrier to organising professional development for teachers. Nine participants revealed that they are supposed to organise professional development for teachers; however that they have not been provided funds to support such an activity. It also emerged that the GES organises professional development in various disciplines, but that they do not make such arrangements for inclusive education. Interestingly, four participants related that teachers will not participate in professional development if they will not get any financial gain. As the GES organises professional development without giving funds to teachers, teachers decline its invitations:

‘The office usually organizes in-service training programs for teachers, but when it comes to inclusive education they will tell you there is no money. We have been writing to them to organize at least one every term, but they are yet to respond to our request.’ (Male, TA7, Ray)

‘When you organize the training, the teachers want incentives [money] before they can take part in the programs. That’s money for their transportation, refreshment, etc. But we don’t have the money to provide the teachers with what they want, since no one give us additional funds to undertake such programs. Because of that, whenever we invite teachers to our programs, they were not coming.’ (Female, TA1, Abena)

While some TAs (*n* = 3) accused the coordinators of not working because of an absence of resources, the coordinators mentioned that they cannot support TAs that fail to perform their duties. This is as a result of the fact that they have not been given resources to perform their activities. Regarding supporting students with disabilities, four participants mentioned that they have chosen subjects to teach so as not to stay idle. Generally, all the participants expressed their frustration with their work because of an absence of materials to work, and some intimated that they were thinking of quitting their job:

‘This work has no future. I even want to stop and move to a special school. I think I would be able to use my skills effectively in a special school than being here, where there is not much work to do. My service is needed most in a special school, where there are resources and materials to support our work.’ (Female, TA1, X)

‘They started the piloting more than five years ago, and no one from the headquarters have come down to see how they are faring. The resource teachers are leaving the profession one after the other. Their presence alone is not enough, as they need logistics to support their work. If you are someone who has passion to work, you would leave the profession.’ (Female, coordinator 5, Dora)

### Intentions

There were positive intentions of participants towards including students with disabilities in secondary schools. Many participants (*n* = 5) mentioned that participation of students with disabilities in secondary school education will enable them to acquire relevant skills for employment and inclusion in a society. Some mentioned that the society has made barriers for persons with disabilities, and that, as such, unrestricted access to secondary schools for them will improve the understanding of disability and will promote acceptance of such persons by members of the society. However, when asked about their preparedness to assist students with disabilities in secondary schools, many said that they do not work to that level. Although a few TAs are able to work in junior secondary schools, none confirmed working in senior secondary schools. While the TAs said that they have not been told to work in secondary schools, the special educators revealed that secondary schools do not accept students with disabilities, hence their decision to limit themselves to primary schools:

‘I wish I could extend to secondary schools. The chance is not there for us to work to that level. They have not given us the permission to work there. They only told us to work in primary schools, but I have maneuvered to work as a resource teacher and teach in a junior secondary school at the same time.’ (Male, TA6, Kofi)

‘For secondary schools, we don’t go there. When officers from Accra [the national capital] came down to introduce the program, they didn’t invite teachers in secondary schools. They called teachers in basic schools (year 1 to 9), so we thought secondary schools are not part of the implementation of the policy.’ (Male, coordinator 9, Abu)

Three participants who had attempted to extend their services to senior secondary schools revealed that teachers and principals prevent them from working. In fact, three other participants also reported that authorities at the GES are scared to approach principals to discuss the idea of practising inclusive education in their schools:

‘I attempted once, and the teachers didn’t cooperate. They told me I can’t come and supervise them. It wasn’t a pleasant experience, so I informed the coordinator about the situation, and he told me not to go there again.’ (Male, TA3, Emma)

‘Education office [*the GES*] is afraid to approach head teachers and discuss with them to accept students with disabilities. I quite remember I discussed with the regional director that we need to create a disability unit in one secondary school. She said, “Eeh! How could they accept such an idea?”’. (Female, coordinator 5, Dora)

Five participants revealed that the ratio of special educators per school is too large, which also makes it impossible for them to work in secondary schools:

‘They have assigned them to cluster of schools, where one person is in charge of about 10 to 15 schools. So what supportive services are they giving to students with disabilities in all schools? As a resource teacher, they have to be in one school and make sure that the children understand everything the teacher is teaching. Meanwhile, they are in school A, school B is teaching, school C and all the schools under them are teaching at the same time. Where do they go?’ (Female, coordinator 5, Dora)

‘I’m supposed to work in more than 10 schools, but due to logistical constraints I have been forced to work from one school only. I don’t have the means to move from one school to another, so have decided to stick to a single school.’ (Female, TA8, Rose)

Participants (*n* = 5) who had experienced supporting students with disabilities in junior secondary schools revealed that students with disabilities are unable to participate in lessons. They mentioned that the curriculum is rigid, and that teachers have to ‘race with time’ to complete the syllabus. Four participants mentioned that teachers are assessed based on the number of exercises they have completed in a week, which makes it difficult for them to include students with disabilities in their lessons. In their view, the teaching of students with disabilities at the basic school level is ineffective, which makes it impossible for these students to progress to a higher level of education.

## Discussion

In this study, Ajzen’s (1991) TPB was adopted as the framework to understand the intentions of special educators regarding supporting inclusive practices in secondary schools. The practice of inclusive education seems to be inextricably linked to the availability of special educators in schools to assist the teaching of students with disabilities. In this study, although attitude seems to be increasing, as opportunities are being created for the participation of children with disabilities in education, there is more room for improvement. According to the participants, teachers continue to recognise their role as vital to successful practice of inclusive education. Unfortunately, in the absence of special educators, teachers appear to struggle to teach students with disabilities. Indeed, the claim of teachers not having skillset to teach students with disabilities is not new as this has been reported consistently in the literature (De Boer et al. 2011; Forlin & Chambers 2011; Mantey 2017; McKay 2016). The inability of teachers to support the teaching of students with disabilities could be linked to the quality of training they receive in inclusive education during pre-service education (Nketsia & Saloviita 2013; Nketsia et al. 2016). There is a possibility of teachers being provided theoretical training in inclusive education without much hands-on practical training. Consequently, teachers may support the idea in principle, however, would struggle to teach the students with disabilities in classrooms. In effect, the implementation of inclusive education at all levels of education would be a *political rhetoric* without much effort being put in place to promote the learning of all. This finding probably calls for more discussion in terms of the skills required by teachers to enable them to teach students with disabilities in classrooms.

Under intentions, scope of practices and job description were found to be barriers to practising inclusive education in secondary schools. Specifically, it emerged that the participants are generally limited to working in primary schools. This finding is surprising because inclusive education was introduced in Ghana to encourage the participation of students with disabilities at all levels of education (Republic of Ghana 2015). Thus, limiting the services of special educators to primary schools could suggest that policymakers might not expect students with disabilities to access post-primary school education. This finding may be attributed to the negative perceptions regarding persons with disabilities in Ghana, as well as the limited knowledge about the capabilities of such persons (Anthony 2011; Mantey 2017; Opoku et al. 2019). Traditionally, persons with disabilities have been described as a liability, and at the family level, little attempt has been made to encourage their participation in productive activities in a society (Opoku et al. 2019). Policymakers might have been influenced by such cultural stereotypes and might not have considered assigning special educators to secondary schools to support the education of students with disabilities in the classroom. This could lead to a situation where students with disabilities will not receive the necessary teaching and learning services in secondary schools. This could limit the prospect of getting many persons with disabilities into higher levels of learning, and ultimately into influential positions. In order for Ghana to increase the prospect of successful participation of students with disabilities in secondary schools, (re)deployment of special educators should be seriously considered.

The job description of the participants seems to have had an adverse impact on their intentions towards supporting the implementation of inclusive education. Effective working relationships between special educators and teachers have been suggested as facilitating inclusive practices (Lyons et al. 2016). However, in this study, some participants stated that their attempts to extend their services to secondary schools are resisted by teachers and school leaders. This finding is partially consistent with the results of previous studies, which have reported that poor communication and a lack of definition of the roles of teachers and special educators create tensions, as both parties play overlapping roles, which disrupts the smooth practice of inclusive education (Rubie-Davies et al. 2010; Whalley 2018). The seemingly negative attitudes of secondary school teachers towards practising inclusive education can be attributed to a lack of engagement between stakeholders regarding inclusive education, as well as a lack of clarity on the role of the participants. It should be reiterated here that secondary school education in Ghana is highly competitive and merit based, and that progression of students is based on their passing examinations set by an external body (Opoku et al. 2021a). Apparently, for maintaining a competitive advantage over other schools, as claimed by the participants, teachers in some schools will not accommodate students with disabilities, who, in their view, cannot excel in external examinations. Also, as emerged in this study, the secondary school curriculum in Ghana is packed, and teachers barely have time to complete all their lessons and prepare students for examinations. In such a system, if there is no communication on the mode of assessment of students with disabilities and if there is no consensus between key stakeholders on inclusive practices, teachers and principals may harbour negative attitudes towards inclusive practices. This finding probably calls for more dialogue between educators on the secondary school curriculum and on which mode of assessment should be used for students with disabilities.

The perceived low self-efficacy of the participants is evident from the study results. The presence of special educators in schools enables students with disabilities to receive the necessary teaching services in the classroom (Hedegaard-Soerensen et al. 2018; Lyons et al. 2016). In particular, the presence of special educators could improve teachers’ confidence, as they have experts to complement their efforts. In this study, the participants mentioned that they have been deployed to work in a number of schools, and that they struggle to assist all the students and teachers. Specifically, almost all the participants are unable to visit all the classrooms in the cluster of schools where they are expected to work. This finding is partially consistent with previous studies, which have found that limited numbers of special educators are a barrier to enacting inclusive education (Opoku et al. 2015). In this study, it appears that the job description of participants is too broad and is difficult to achieve. It is reasonable to point out that the form and scope of involvement of special educators in the implementation of inclusive education in Ghana seem to be different from the international best practices mentioned in the literature. In some contexts, special educators are expected to work in schools as advocates of inclusive education, while TAs will work closely with classroom teachers. However, according to the Ghanaian model described here, special educators working as TAs claim that they have been assigned to work in a cluster of schools. It is unsurprising that many schools and teachers do not receive assistance from special educators in the classroom (Opoku et al. 2021a, 2021b). Because of the broad scope of their responsibilities, the participants may be stretched, and as such, they may have low self-efficacy to support inclusive practices in secondary schools. It is necessary that policymakers reconsider deploying special educators to enhance inclusive practices.

One of the major factors that has an impact on the self-efficacy of teachers is the absence of finances and teaching materials. The unavailability of these resources was blamed on the failure of subjective norms (the government). Although school leaders were mentioned by some participants as one of the parties that do not make funds available, it is apparent that ultimately the responsibility lies with the government and its agencies to ensure that vital resources are made available to the participants. This finding is consistent with that of previous studies, which have reported that a lack of funds, teaching materials and recognition have contributed to TAs being dissatisfied with their job and being unable to improve the learning of students with disabilities (Al-Natour et al. 2015; Butt 2016; Devecchi et al. 2012; Sharma & Salend 2016; Timberlake 2018). As is repeatedly mentioned in the literature, posting professionals such as special educators in schools and changing teachers’ attitudes may not be sufficient to ensure the success of inclusive education (Ahmmed et al. 2014). There should be requisite teaching materials as well as planned professional development training, so as to ensure that the school community has access to appropriate knowledge in order to enact inclusive education (Ainscow & Sandill 2010). The participants’ expression of frustration is expected, because they have been deployed to work without the necessary tools. The inability of participants to access resources has contributed to their low self-efficacy and their desire to leave the profession or to perform other duties rather than supporting students with disabilities. It has also contributed to them being unable to function as expected. The government could consider budgeting for inclusive education in order to enable special educators to have access to the needed resources to work.

The limited support from the subjected norm was discussed by the study participants. One of the approaches to practising inclusive education is a system approach, where various stakeholders play a contributory role in school practices (Ainscow & Sandill 2010; Lyons et al. 2016). While the government seems to have reneged on its commitment to provide the needed resources, as mentioned by the participants, one would have expected parents to prioritise the learning of their children with disabilities. Parents have a role to play to help their children with disabilities succeed in regular classrooms. However, in this study, the participants stated that support from subjective norms such as parents is not available. Specifically, the participants claimed that parents are unable to purchase the needed materials and food, to prepare their children for schools or to honour invitations to discuss the welfare of their children. Poverty was mentioned in this study as one of the reasons for parents’ inability to support their children. This finding is not surprising because poverty has been found to affect not only persons with disabilities but also other members of the family (Dogbe et al. 2019; Opoku et al. 2017). With limited assistance from the government to parents (Opoku et al. (2021a), it is likely that the latter may have inadequate finances to support the education of their children with disabilities, which might, in turn, affect the practice of inclusive education. There is the possibility that parents might lack understanding of inclusive education, and that, as such, they are unmotivated to support or invest in the education of their children with disabilities (Opoku et al. 2019). This probably calls for more engagement between stakeholders, including parents of children with disabilities, on their contribution towards successful practice of inclusive education.

## Study limitations

There are several limitations in this study, which need to be addressed by future studies. Firstly, it was beyond the scope of this study to verify assertions made by the study participants, such as the assertions that students with disabilities are participating effectively, that support from parents is limited, and that there is a lack of teaching materials. Including the voice of officials at the SPED, the GES and the Ministry of Education in the study could have clarified some of the claims made by the participants. It is important to state here that the participants that took part in this study are employed specifically to oversee the implementation of inclusive education in Ghana. This means that their accounts may be a true reflection of what pertains on the ground. Notwithstanding, it is recommended that future studies explore the perceptions of policymakers, parents and students with disabilities regarding inclusive practices in secondary schools. Also, it is unclear whether education policies limit the implementation of inclusive education in primary schools. Future research could analyse education policy documents and ascertain the level of commitment towards promoting equitable access to all levels of education.

## Conclusion and recommendations

As part of global efforts to eradicate extreme poverty among vulnerable groups, governments have been urged to extend universal access to education from primary schools to secondary schools to enable the participation of all students (United Nations 2015). It is believed that graduates from secondary schools will be mature enough and will even acquire skills to participate in economic activities in a society. Using Ajzen’s (1991) TPB as the theoretical framework, the intentions of special educators to assist teachers and students with disabilities in secondary school classrooms in Ghana were explored. In terms of attitudes, there seems to be a gradual understanding of inclusive education. However, there are inherent challenges preventing successful implementation of inclusive education in secondary schools in Ghana.

Unlike the case in previous studies, where TAs did not have the requisite qualifications (Butt 2016; Butt & Lowe 2012), in this study the participants are qualified special educators; however, they are unprepared to extend their services to secondary schools because of the following reasons: their job description, which limits them to primary schools, resistance from secondary school teachers and principals, lack of support from stakeholders, and inadequate financial and material resources to effectively execute their functions. These challenges affected the intentions to support inclusion in secondary schools, and contributed to self-efficacy and helplessness because of lack of commitment from subjective norms. Without the presence of the study participants, such students may be excluded and denied their right to secondary education.

The services of special educators have been identified as vital to the success of inclusive education (Devecchi et al. 2012; Hedegaard-Soerensen et al. 2018). This underscores the need for policymakers to address the concerns raised by the participants who took part in this study. Firstly, the government may consider providing the needed resources and facilities to implementers, such as special educators, in order to enable them to promote the learning of students with disabilities. This could be achieved through budgetary allocations to schools to enable them procure the teaching materials needed to support the teaching of students with disabilities. This could enhance the self-efficacy of teachers and encourage them to discharge their duties. Secondly, the SPED may reconsider deploying special educators to clusters of schools. It appears that the strategy being used may affect the ability of special educators to make an impact in schools. Policymakers may consider deploying special educators to every school. For example, if each secondary school has its own special educator, it could be a useful start to making their presence felt in schools and classrooms. Thirdly, there is a need for discussion between stakeholders such as teachers, teacher educators, special educators, persons with disabilities and parent on the relevant skills required by teachers to enable them practise inclusive education. The deliberation could extend to using evidence-based research to guide the reformation of inclusive education curriculum for training teachers. Moreover, SPED could organise sensitisation programmes on the implementation of inclusive education regularly for the community, school leaders, teachers and parents. This would help them to understand the implementation of inclusive education and to collaborate with special educators to facilitate inclusive practices.

## Appendix 1

### Interview guide scheduled for special educators.

1. Could you please tell me about your career in education?
2. How do you understand the term ‘disability’?
3. What is your view on the inclusion of students with disabilities in regular classrooms?
4. What terms are currently used in your school to describe different learners?
5. How do you see the implementation of inclusive education in this school?
6. Tell me about your role as special educator/TA in the classroom.
7. Tell me about the students and subjects you support.
8. Can you share with me some of the ways that you provide support to the teachers and students?
9. What is your working relationship with teachers?
10. What do you think about collaboration between teachers and special educator/TAs?
11. How are you involved in the preparation of lessons?
12. How do you prepare to support students who are learning different subjects from more than one teacher?
13. How flexible are the lessons to enable the participation of students with disabilities?
14. How do teachers involve students with disabilities in teaching and learning in the classroom?
15. What are some of the resources used in teaching and learning activities?
16. Do you face challenges supporting students in specific subjects?
17. What are some of the challenges you encounter in your day-to-day teaching activities?
